# Highly-Accelerated Real-Time Cine MRI using compressed sensing and parallel imaging

**DOI:** 10.1186/1532-429X-13-S1-P25

**Published:** 2011-02-02

**Authors:** Li Feng, Ricardo Otazo, Monvadi B Srichai, Ruth P Lim, Daniel K Sodickson, Daniel Kim

**Affiliations:** 1 New York University School of Medicine, New York, NY, USA

## Purpose

To develop and evaluate highly-accelerated real-time cine MRI using compressed sensing and parallel imaging.

## Introduction

Breath-hold cine MRI with balanced steady-steady free precession (b-SSFP) may yield non diagnostic image quality in patients with impaired breath-hold capacity and/or arrhythmias. In such patients, it may be necessary to perform real-time cine MRI. Currently, dynamic parallel imaging methods, such as TSENSE [1] and TGRAPPA [2], can be used to achieve only moderate acceleration rates (R) of 2-3 using standard body and spine coil arrays. We propose the application of a recently developed joint acceleration technique (CS-PI)[3] that combines compressed sensing [4] and parallel imaging for highly-accelerated, real-time cine MRI with clinically acceptable spatiotemporal resolution.

## Methods

Real-time cine MRI pulse sequences with b-SSFP readouts and TGRAPPA and CS-PI accelerations with R=4 and R=8 were implemented on 3T whole-body MRI scanners (Siemens; Tim-Trio & Verio) equipped with standard body and spine coil arrays (12 elements total). The relevant imaging parameters include: FOV=320mm x 320mm, acquisition matrix size=128x128, TE/TR=1.37/2.7ms, receiver bandwidth=1184 Hz/pixel, and flip angle=40^o^. The temporal resolutions were 86.4, and 43.2 ms for R = 4 and 8, respectively. Seven patients (mean age=41.5±20.7 years) undergoing clinical CMR were imaged in mid-ventricular short-axis and long-axis planes, following completion of the clinical examination using free breathing and electrocardiogram gating. The cine data sets were randomized and blinded for qualitative evaluation (image quality, artifact, noise; 1-5; lowest-highest) by a cardiologist and a radiologist. Statistical analysis was performed to compare the mean scores between the 4 groups (TGRAPPA-R4, TGRAPPA-R8, CS-PI-R4, CS-PI-R8) and between each pair of groups.

## Results

**Figure 1:**
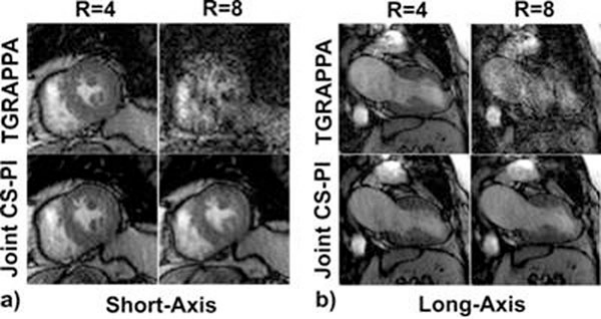
a) Short-axis view and b) long-axis view. For each view: (top row) TGRAPPA; (bottom row) joint CS-PI; (left column) R=4; (right column) R=8.

Figure 1 shows images of end-systolic frames in mid-ventricular short-axis and 2-chamber views. According to the Kruskal-Wallis test, the 4 groups were significantly different (p0.05): image quality, CS-PI-R4 vs. CS-PI-R8; artifact, CS-PI-R4 vs. CS-PI-R8; noise, TGRAPPA-R4 vs. CS-PI-R8, TGRAPPA-R4 vs. CS-PI-R4, CS-PI-R4 vs. CS-PI-R8. These preliminary results suggest that TGRAPPA can yield robust results at R=4, whereas CS-PI can yield robust results up to R=8.

## Discussion

This study demonstrates the feasibility of performing highly-accelerated real-time cine MRI using a joint CS-PI technique. An 8-fold accelerated real-time cine MRI protocol can achieve spatial resolution of 2.5mm x 2.5mm and temporal resolution of 43.2 ms, with adequate image quality. This accelerated protocol may be useful for debilitated patients with reduced breath-hold capacity and/or arrhythmias for rapid left ventricular functional evaluation.

**Figure 2:**
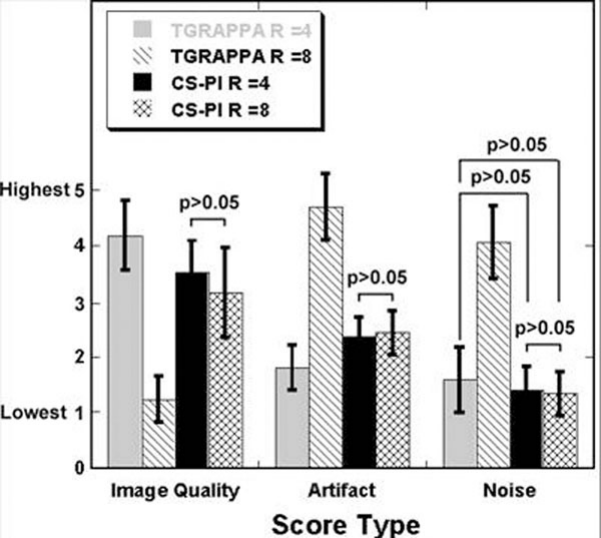
Plots of mean scores: (left) image quality, (middle) artifact, and (right) noise. Pairs with no significant difference are noted with p>0.05.
